# Qualitative Analysis of One Phase II Clinical Trial for the Drug Treatment of Large-Area Cerebral Infarction

**DOI:** 10.3390/life16040666

**Published:** 2026-04-14

**Authors:** Zhongzheng Han, Yanchao Li, Huibin Zou

**Affiliations:** Department of Pharmaceutical Engineering, College of Chemical Engineering, Qingdao University of Science and Technology, Qingdao 266042, China; 17692152832@163.com (Z.H.); yanchaoli@mails.qust.edu.cn (Y.L.)

**Keywords:** clinical trial, large-area cerebral infarction, quality control, monitoring

## Abstract

**Purpose:** Quality evaluation data for clinical trials of large-area cerebral infarction are lacking. The objective of this study was to identify critical quality control issues specific to clinical trials in large-area cerebral infarction and to propose and evaluate corresponding strategies for the improvement of related clinical trials. The findings aim to inform and enhance quality management practices in future trials within this clinical area. **Methods:** Using one phase II clinical trial for the drug treatment of large-area cerebral infarction as the research model, we analysed the quality control issues in the participating centers. In the primary survey, quality issues were systematically examined from one key center (center A) and 26 additional centers participating in the trial. On the basis of the quality issues identified in the primary survey, six preventive measures were developed for these quality issues. These measures were subsequently applied in a follow-up secondary survey at two key centers (center A and center B); however, the other 53 centers were not subjected to the implementation of the measures. Chi-square analysis was conducted to evaluate the effectiveness of the corresponding preventive measures. **Results:** In the primary survey, no statistically significant differences were observed in the incidence rates of issues between center A and other centers, with the exception of PD-related quality issues. Following the implementation of the preventive measures, chi-square analysis revealed a statistically significant reduction in AE-related issues at center A compared with the other centers (*p* < 0.05). The improvement in AE-related issues represents the most notable outcome of this study. The direct comparison within center A revealed a reduction in quality issues per subject from 9.42 in the primary survey to 5.93 in the secondary survey. In addition, the number of AE-related issues decreased from 4.58 per subject to 2.20 per subject. **Conclusions:** The results of this study suggest that preventive measures are feasible for improving quality control in large-area cerebral infarction clinical trials. However, these quality measures require broader validation through larger-scale clinical trials.

## 1. Introduction

Drug clinical trials are systematic investigations conducted with human subjects—either patients or healthy volunteers—to evaluate the efficacy, safety, pharmacological characteristics, and risk–benefit profile of investigational drugs. As the means for obtaining core evidence supporting drug registration and marketing authorization, clinical trials play a decisive role in confirming therapeutic benefits and identifying potential adverse reactions [1,2,3].

Quality control (QC) in clinical trials encompasses technical measures and operational procedures implemented within an overarching quality assurance framework to ensure that all trial-related activities comply with predefined protocols, regulatory requirements, and good clinical practice (GCP) standards [4,5,6].

In recent years, international regulatory agencies have paid more attention to risk-based quality management. In 2013, the U.S. Food and Drug Administration (FDA) issued guidance on a risk-based approach to monitoring clinical investigations [7]. This was followed by a reflection paper released by the European Medicines Agency (EMA) on risk-based quality management in clinical trials [8]. Both guidelines highlight that risk-based quality management is a continuous and iterative process, underscoring the essential role of QC in ensuring trial robustness.

Particularly for acute conditions such as large-area cerebral infarction—characterized by sudden onset, rapid progression and high mortality rates—the implementation of clinical trials imposes more stringent demands on quality control measures.

Large hemispheric infarction (LHI), a severe subtype of ischaemic stroke, imposes particularly stringent demands on clinical trial implementation owing to its abrupt onset, rapid progression, and extremely high mortality. According to the Global Burden of Disease (GBD) 2019 database, stroke remains the second leading cause of death worldwide. Ischaemic stroke accounts for 62.4% of all stroke cases, representing 7.63 million incident events and 3.29 million deaths in 2019 [9]. By 2030, the number of ischaemic stroke-related deaths is projected to increase to 4.9 million [10,11,12,13]. China has the highest lifetime risk of stroke globally [14,15,16,17,18], and with an increasing ageing population, the incidence, prevalence, and associated disease burden are expected to continue to increase [19,20,21].

LHI, also referred to as massive cerebral infarction, is often complicated by malignant cerebral oedema, a life-threatening condition for which mortality increases to 40–80% [22,23,24,25]. These data highlight the urgent need to develop novel therapeutic strategies for LHI.

Current interventions for LHI-associated cerebral oedema—such as mannitol and hypertonic saline therapy, as well as decompressive craniectomy—primarily target established oedema but lack efficacy in preventing oedema onset [26,27,28,29,30]. Given the scarcity of effective pharmacological therapies and the high mortality associated with malignant cerebral oedema, LHI represents a compelling and unmet clinical need, making it a potentially important direction for future drug development [31].

Currently, antitumour drugs constitute the therapeutic area attracting the greatest global investment in pharmaceutical R&D. Quality control in oncology trials is widely regarded as particularly challenging because of long study durations, heterogeneous disease trajectories, high incidences of comorbidities, frequent adverse events, concurrent medications, and complex efficacy assessments. Nevertheless, oncology trials typically feature flexible visit schedules—often every 2–4 weeks—which allows site personnel sufficient time to process and verify clinical data [32].

In contrast, clinical trials for LHI must contend with abrupt onset, a rapidly evolving clinical status, high mortality, frequent comorbidities, intensive monitoring, and a dense schedule of early visits.

Overall, compared with oncology trials—in which workload is distributed more evenly across a longer study duration—LHI clinical trials exhibit a concentrated workload peak and greater operational intensity at the execution stage. Therefore, from a quality control perspective, LHI trials pose more formidable challenges and warrant heightened attention to ensure data integrity, protocol adherence, and subject safety.

The aim of this study was to analyse quality issues identified in an initial survey of large-area cerebral infarction clinical trials and then develop corresponding preventive measures and evaluate their effectiveness in subsequent trials.

## 2. Methods

### 2.1. Clinical Trial Methods in the Primary Survey

The clinical trial in the primary survey was a phase II, randomized, double-blind, placebo-controlled, registrational clinical trial, with the specific indication of treating severe cerebral oedema following large hemispheric infarction. The trial involved 27 centers in China with a target enrolment of 40 subjects. The trial was approved by the National Medical Products Administration (NMPA) (Approval No.: 2021LP01923) and received ethics approval from the institutional review boards of all 27 participating centers.

The trial employed an experimental drug group and a placebo group. The dosing regimen involved an initial intravenous bolus, followed by a continuous intravenous infusion for more than 72 h. The visit schedule included the screening period, D1, D2, D3, D4, D5-D6, D7, D14, D30, and D90, with visits being notably (intensive) within the first four days after enrolment. The key inclusion criteria were as follows: (1) age 18–80 years, regardless of sex; (2) no significant prestroke disability, as judged by the investigator (mRS < 3); (3) National Institutes of Health Stroke Scale (NIHSS) score ≥ 10 at screening; (4) LHI defined, in order of priority, as follows: (a) infarct volume of 80–300 cm^3^ measured by means of magnetic resonance diffusion-weighted imaging (MRI-DWI) or (b) core infarct volume of 80–300 cm^3^ measured by means of computed tomography perfusion (CTP); if the results from these imaging modalities were inconsistent for a given subject, the investigator was required to make a reasonable judgement on the basis of a comprehensive assessment of all available information (e.g., scan timing and the imaging method best reflecting infarct size) and document the rationale; and (5) for subjects with a clearly defined time of onset, the time from onset to study drug administration was required to be ≤10 h; if the time of onset was unknown, administration was required within 10 h of the time the subject was last known to be well.

Considering criteria 3 and 4 together, the eligible subjects in the primary survey trial were predominantly patients with severe acute ischaemic stroke. Furthermore, the 10-h time window from onset to drug administration specified in criterion 5 posed greater challenges for clinical trial sites in terms of providing informed consent, screening assessments, and medical review in a timely and thorough manner after patient arrival.

### 2.2. Qualitative Analysis of the Primary Survey

In the primary survey, the author systematically analysed the qualitative issues documented at a pivotal center—Center A—as well as the 26 other participating centers. The following issues were assessed: (1) clinical trial process documentation; (2) adverse events; (3) protocol deviations; (4) electronic data capture/electronic case report form; and (5) other issues. These categories were established to evaluate the effectiveness of these measures through statistical analysis of the frequency of quality issues documented at each center.

The aim of this qualitative analysis was to summarize the root causes of various quality issues and, by incorporating the characteristics of the subjects who met the inclusion/exclusion criteria (including pre-enrolment challenges and potential postenrolment risks), to subsequently propose corresponding preventive measures to control the frequency of these issues.

### 2.3. Clinical Trial Methods of the Secondary Survey

The clinical trial examined in the secondary survey was a phase II/III randomized, double-blind, placebo-controlled, registrational clinical trial; it was a subsequent trial to the one analysed in the primary survey, with the same indication of treating severe cerebral oedema following large hemispheric infarction. This trial was also conducted in China and involved 55 centers with a target enrolment of 725 subjects. The clinical trial was approved by the NMPA (Approval No.: 2021LP01923) and received ethics approval from the institutional review boards of all 55 participating centers.

The investigational drug used in the secondary survey was identical to that used in the primary survey. However, the group allocation differed, consisting of a low-dose experimental group, a high-dose experimental group, and a placebo group. The dosing regimen included an initial intravenous bolus followed by a continuous intravenous infusion for more than 72 h. The visit schedule included the screening period, >0–12 h, 24 h, 48 h, 72 h, 96 h, D7, D14, D30, and D90, with visits similar to those within the first four days after enrolment. The key inclusion criteria were as follows: (1) age 18–80 years (inclusive), regardless of sex; (2) no significant prestroke disability, as judged by the investigator (mRS < 3); (3) National Institutes of Health Stroke Scale (NIHSS) score ≥ 10 at screening; (4) large hemispheric infarction, in order of priority, as follows: (a) infarct volume of 80–160 cm^3^ measured by means of magnetic resonance imaging–diffusion weighted imaging (MRI–DWI) or (b) core infarct volume of 80–160 cm^3^ measured by means of computed tomography perfusion (CTP); if the results from these imaging modalities were inconsistent for a given subject, the investigator was required to make a reasonable judgement on the basis of a comprehensive assessment of all available information (e.g., scan timing and the imaging method best reflecting infarct size) and document the rationale; and (5) for subjects with a clearly defined time of onset, the time from onset to study drug administration was required to be ≤10 h; if the time of onset was unknown, administration was required within 10 h of the time the subject was last known to be well.

Overall, the inclusion criteria for the secondary survey trial remained largely consistent with those of the primary survey trial, except for criterion 4, where the required core lesion volume was revised from 80 to 300 cm^3^ in the primary survey to 80–160 cm^3^ in the secondary survey.

In the secondary survey, the preventive measures were intentionally limited to several centers to assess their feasibility and effectiveness prior to broader application. Centers A and B were designated pivotal centers and implemented predefined preventive measures, while the remaining 53 centers served as the control group. Five qualitative issues were analysed between the testing and control groups. A self-controlled concurrent study was conducted at center A to compare the number of quality issues across categories in trial A with those in another large-area cerebral infarction clinical trial conducted concurrently at the same center (trial B) to assess the presence of learning effects. Finally, a problem traceability flow model was constructed to perform a cluster quantitative analysis of quality defects occurring throughout the full trial cycle.

### 2.4. Data Analysis

The data were derived from monitoring reports and audit reports generated during the trial. The use of these data was approved by the sponsor, Beijing Shengdi Pharmaceutical Co., Ltd. (Beijing, China), and all the data were anonymized prior to analysis to protect patient privacy.

CRAs and investigators are involved in assessing qualitative issues and developing corresponding quality control measures. Chi-square tests were used to assess statistically significant differences in issue incidence between center A and other centers in the primary survey and between centers A/B and the remaining centers in the secondary survey. Quantitative statistics (frequency, percentage, and mean issues per subject) were also employed to compare trends in issue incidence at center A across the two surveys as well as trends in issue incidence in the self-controlled concurrent study at center A.

## 3. Results

### 3.1. Analysis of the Quality Challenges in Large Hemispheric Infarction Clinical Trials

#### 3.1.1. Major Categories of Quality Issues at Center A

In the initial phase II trial examined in this study, center A enrolled a total of 12 subjects during the trial period. This center underwent 11 monitoring visits and 2 audits, which resulted in the documentation of 113 issues. The average number of issues recorded per subject was 9.42, as shown in Table 1.

Within these categories, the primary concerns centerd on AEs and PDs, which collectively accounted for 81 issues (71.7% of the total).

#### 3.1.2. AE Issues at Center A

AE issues (*n* = 55) were primarily distributed across three areas:

Clinical significance assessment of abnormal findings (*n* = 21)—mainly included failure to assess clinical significance and inconsistent assessment criteria;

Underrecording and underreporting of AEs (*n* = 30)—primarily involved missed recording and reporting of AEs;

Rationality of AE naming/grading and consistency of documentation (*n* = 4)—mostly concerned unrecorded changes in the grade of AEs and inconsistencies between source documents.

Furthermore, the rationale for determining drug-relatedness was problematic in some cases, where AEs were deemed definitely unrelated to the investigational product without supporting notes.

#### 3.1.3. PD Issues at Center A

PDs (*n* = 26) were primarily related to missed examinations and window violations, predominantly during the screening period. The protocol required dosing within 10 h of onset, but most critically ill patients could not provide urine samples, resulting in incomplete screening assessments for nearly all 12 subjects. This led to 14 deviations (53.8% of all deviations).

Additionally, 8 deviations (30.8%) occurred because the clinical team was unfamiliar with the protocol, leading to failure to repeat the required tests. Another 4 deviations (15.4%, involving 2 subjects) occurred because rapid disease progression necessitated transfer to other departments, where protocol-required assessments were difficult to perform within the specified windows.

Other issues included the incomplete documentation of eligibility criteria in medical records, the improper storage of source documents, and EDC entry errors.

#### 3.1.4. Analysis of Quality Issues at Center A

Subjective factors contributing to these quality issues included the following:

The lack of the ability of some investigators to properly assess and document abnormal cases during clinical research;

CRC workload constraints due to concurrent involvement in other studies, potentially compromising protocol adherence;

Unrealistic protocol requirements, particularly the 10-h dosing window, preventing the completion of all screening assessments before enrolment, inevitably causing deviations; 

Challenges in maintaining protocol compliance when subjects were transferred to non-research departments.

#### 3.1.5. Comparative Analysis of Quality Issues Between Center A and the Other Centers

In this clinical trial, the remaining participating centers enrolled a total of 28 subjects. A total of 67 monitoring reports and 2 audit reports were collected, documenting 458 issues. The average number of issues per subject was 16.36. The distribution of issues by category is presented in Table 1.

Compared with center A, the other participating centers had a higher number of issues per subject. However, the issues were similarly concentrated in the areas of AEs and PDs, which collectively accounted for 381 issues, representing 83.2% of the total.

Given the relatively low frequency of issues related to clinical data source verification, subject screening and eligibility, SAE/SUSAR/AESI recording and reporting, management and documentation of biological samples, and monitoring, these five categories were consolidated into an “Other” category for analysis. Consequently, five quantitative indicators were selected for evaluation: CTPD, AEs, PDs, EDC/eCRF, and Other. The average number of issues per subject was calculated separately for each of these categories.

**Figure 1:**
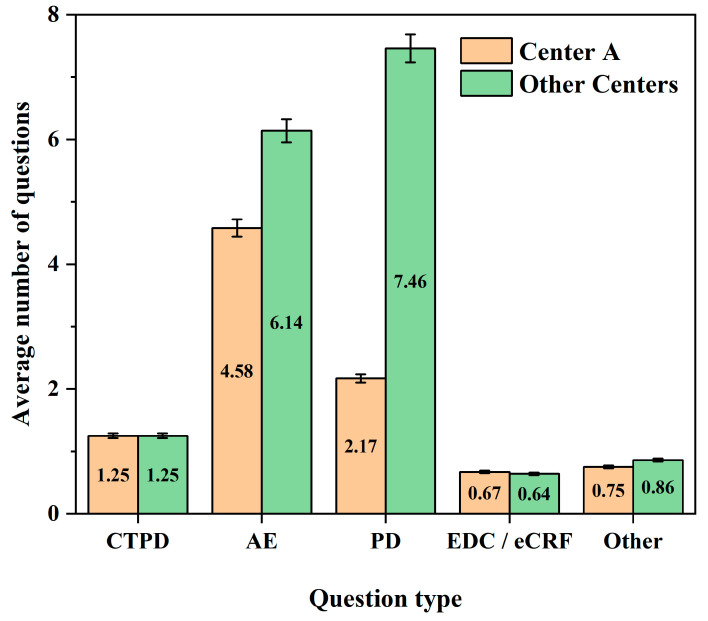
The average number of quality issues per subject by type in the primary survey. CTPD: clinical trial process documentation; AE: adverse event; PD: protocol deviation; EDC/eCRF: electronic data capture/electronic case report form.

As shown in the Figure 1, the number of issues per subject for the CTPD, EDC/eCRF, and Other categories was comparable between center A and the other centers. However, the other centers had a slightly higher number of issues related to AEs and a substantially greater number of PDs.

#### 3.1.6. Subject Characteristics in the Primary Survey

An analysis of the core elements of the clinical trial in the primary survey revealed that the subjects typically presented as emergencies, with the protocol requiring dosing within 10 h of onset. Given that many patients arrive at research departments nearly 10 h after symptom onset, it is essential for both the CRC and investigators to be thoroughly familiar with the protocol to complete the informed consent process and screening assessments within a tight window. Furthermore, given the intensive visit schedule within the first four days post-enrolment and the critical condition of these patients—who are at high risk of deterioration—numerous abnormal laboratory findings may occur during this period, necessitating accurate and complete documentation of adverse events. Last, owing to the high risk of disease progression associated with large hemispheric infarction, patients may require transfer to other departments. Given that the receiving departments are not typically authorized research units, such transfers may increase the risk of protocol deviations.

### 3.2. Quality Control Measures for Large-Hemispheric Infarction Clinical Trials

On the basis of a comprehensive analysis of issues identified across all centers in the primary survey, the following preventive measures were proposed and designed to enhance quality control and reduce the occurrence of problems.

Standardize medical record documentation: A standardized medical record template was designed to provide a unified documentation framework for large-area cerebral infarction clinical trials, aiming to standardize recording practices at the source. The template ensures organized and centralized information by incorporating multiple structured fixed sections. A “carry-forward and update” mechanism was adopted to enable the continuous management of dynamic information such as medical history, adverse events, and concomitant medications. For all examination results, the template mandates the listing of all abnormal values and requires that each be assessed for clinical significance, ensuring the completeness and consistency of abnormality evaluations. Additionally, a “medical record amendments and supplements” section was established to require the unified documentation and traceability of all modifications.

Ensure consistency in clinical judgment: A set of structured measures was developed and implemented. First, during the study initiation phase, it was explicitly agreed that the same investigator would, whenever possible, be responsible for the longitudinal assessment of abnormal findings for a given subject to ensure consistency in evaluation criteria. If a change of investigator was unavoidable, a handover process was established, requiring the original investigator to clearly document prior assessment justifications and key points requiring attention. Second, the consistency oversight function of the CRC was strengthened through specialized training, enabling the CRC to identify discrepancies in the assessment of similar abnormal findings among different investigators and to promptly alert the investigators to document the specific justifications for their assessments. Finally, a closed-loop mechanism for feedback and retraining was established. For sites with recurrent issues related to inconsistent assessment criteria, targeted training sessions were organized, and changes in issue incidence were continuously monitored to ensure uniform implementation of evaluation standards, thereby forming a closed loop for continuous improvement.

Strengthen CRC selection and support: To systematically strengthen the selection, evaluation, and support mechanisms for clinical research coordinators (CRCs), a multilevel structured strategy was established. First, by designing the Stroke Clinical Trial CRC Position Suitability Interview Scoring Form and setting a reference baseline of 60 points for hiring, CRCs were quantitatively assessed and screened in terms of regulatory familiarity, process execution, communication and collaboration, and time management, ensuring basic competency at the entry level. Second, for centers with high enrolment potential, backup CRC support was prearranged in coordination with the sponsor and site management organization (SMO) to establish a dynamic human resource contingency plan, thereby avoiding workload overload due to concentrated enrolment over a short period and reducing the risk of protocol deviations such as visit window violations and missed assessments. On the basis of these efforts, we further developed the Standard Operating Procedure for CRCs in Stroke Clinical Trials, which defines operational steps, quality control checkpoints, and completion timelines for key processes and provides guidance on identifying and correcting common errors. This SOP aims to systematically enhance the standardization and consistency of CRC performance, thereby strengthening the foundation of clinical trial quality at the operational level.

Develop an AE quick reference guide: Referencing CTCAE v5.0, we systematically reviewed common adverse events in stroke clinical trials and developed the Standardized Quick Reference Guide for Common Adverse Events in Stroke Clinical Trials. The guide is presented in tabular format and covers nine major categories of adverse events: adverse haematological/haematological events, adverse blood biochemistry events, adverse coagulation function events, adverse urinalysis events, allergic reactions/infusion-related reactions, worsening primary neurological events, infectious complications, vascular complications, and other complications and functional deficits. For each adverse event, the guide clearly lists grading criteria for grades 1–5, specific descriptions, and relevant laboratory parameters, providing investigators with a unified and standardized reference for assessment. The guide was made available in three formats—portable pocket-sized booklets, wall-mounted key information posters, and an electronic version for on-demand access—to ensure rapid consultation in busy clinical settings. During the study initiation phase, specialized training was provided, along with case-based exercises, to strengthen proficiency in terminology use, grading determination, and identification of serious adverse events.

Establish a clear cross-departmental referral process: A structured accountability and coordination mechanism for subject cross-sectoral referral, centerd on the principles of “group-based assignment, full-process follow-up, and supervisory oversight,” was developed to systematically address coordination issues during the referral process. Prior to study initiation, the principal investigator designated a Primary Sub-I responsible for the overall management of enrolled subjects, as well as for the collaboration of Sub-Is with fixed authorization in referral departments (e.g., rehabilitation, ICU). A dedicated WeChat group was created for each referral department to ensure a fixed channel for information transmission and clear accountability. During the enrolment and execution phase, when the Primary Sub-I decided to refer a subject, the collaborating Sub-I was immediately notified in the WeChat group and confirmed acceptance. The CRC provided reminders in the WeChat group before key time points, and the collaborating Sub-I promptly confirmed completion. In the event of emergencies, the collaborating Sub-I communicated immediately in the WeChat group for joint decision-making. All operations were documented in the WeChat group, forming a traceable closed loop. At the supervisory level, the CRC reviewed WeChat group messages daily and followed up on unconfirmed items, making phone calls if no response was received within two hours.

Establish a targeted monitoring plan: First, an “advanced initial monitoring time point” strategy was implemented, requiring that the first monitoring visit be completed within seven days after enrolment of the first subject. This facilitated the early identification of execution deviations, enabled data correction within the D7 visit window, and reinforced the research team’s awareness of quality from the outset. Second, a “dynamic monitoring frequency” strategy was adopted, adjusting the monitoring frequency on the basis of the enrolment rate of each site. Using a “visit node mapping” approach, monitoring visits were scheduled to coincide with overlapping windows prior to upcoming visits for multiple subjects, ensuring that issues could be identified and corrected within the visit window. This established a real-time “monitoring-execution-feedback” closed loop and prevented the recurrence of similar issues in subsequently enrolled subjects. Finally, the Quick Start Guide for CRAs in Stroke Clinical Trials was developed, focusing on three core modules: basic medical knowledge regarding stroke, guidelines for assessing core scales, and practical monitoring considerations. By making implicit professional knowledge explicit and systematizing fragmented information, the guide facilitated a shift from “experience-based monitoring” to “knowledge-based monitoring,” effectively reducing the quality risks associated with limited professional expertise.

### 3.3. Implementation of Quality Control Measures

On the basis of the experience gained from the previously mentioned primary survey, the preventive measures outlined above were implemented in a subsequent secondary survey led by the author. While the protocol underwent certain modifications, it remained fundamentally consistent with the previous trial, retaining the same investigational product and indication (LHI).

In the subsequent secondary survey, the author was responsible for two key centers: one overlapping with the previous study (center A) and one newly initiated center (center B). These two centers implemented quality prevention measures, while the remaining 53 centers served as non-implementation controls. The efficacy of these measures was evaluated through statistical analyses of issue frequency data.

### 3.4. Chi-Square Test of Issue Frequency in the Primary Survey

A chi-square test was conducted to compare the incidence of five categories of quality issues—CTPD, AE, PD, EDC/eCRF, and Other—between center A and the other centers (Table 2).

The analysis revealed no statistically significant differences in the incidence of CTPD, AEs, EDC/eCRF, and other issues between the two groups (all *p* > 0.05). In contrast, PD-related issues were significantly more frequent in the other centers (χ^2^ = 10.292, *p* = 0.001).

A detailed review revealed that this difference was driven primarily by localized clusters of poor subject management at several centers. Three centers had six subjects who each accumulated ≥10 protocol deviations, primarily because of window violations, missed assessments, missed visits, delayed SAE reporting, and one instance of unauthorized drug discontinuation.

For example, a single subject with 12 deviations experienced five window violations, three missed assessments, two missed visits, one late SAE report, and one unauthorized treatment interruption. These findings highlight the substantial operational challenges inherent to stroke clinical trials and underscore the importance of early, continuous oversight.

Overall, the primary survey demonstrated comparable quality performance between center A and the other centers in four of the five issue categories, with PDs emerging as the only category with statistically significant between-center differences.

### 3.5. Comparison of Quality Outcomes Before and After Preventive Measures in the Subsequent Secondary Survey

**Figure 2:**
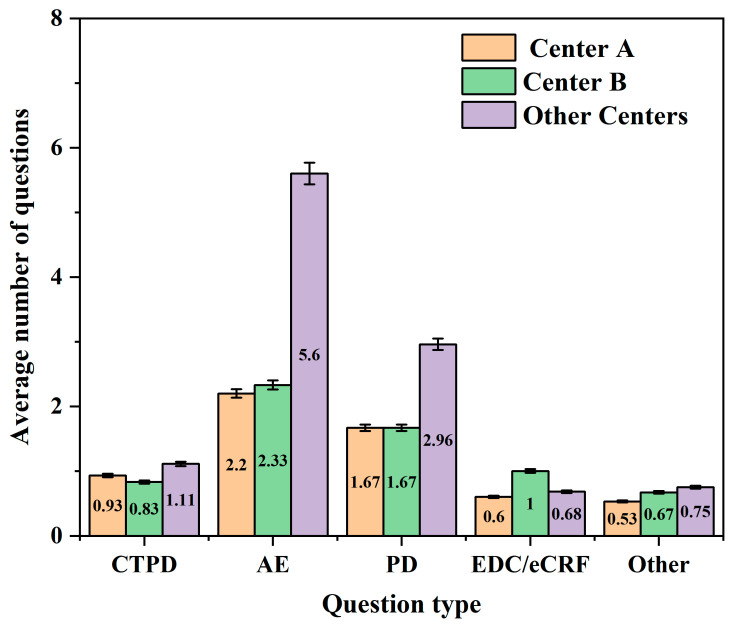
The average number of issues per subject by type in the subsequent secondary survey. CTPD: clinical trial process documentation; AE: adverse event; PD: protocol deviation; EDC/eCRF: electronic data capture/electronic case report form.

In the subsequent secondary survey, centers A and B implemented predefined preventive quality control measures. At center A, 15 subjects were enrolled, and 89 issues were documented (5.93 issues/subject), whereas at center B, 6 subjects were enrolled, and 39 issues were documented (6.50 issues/subject). In contrast, at the other centers, 84 subjects were enrolled, and 932 issues were recorded (11.10 issues/subject). To enable consistent comparisons across centers, five analytic categories were maintained (CTPD, AE, PD, EDC/eCRF, and Other). The average number of issues per subject by category is presented in Figure 2.

Compared with the other centers, both center A and center B had lower issue frequencies across nearly all categories. The only exception was a slightly greater number of EDC/eCRF issues at center B, likely reflecting factors such as staff inexperience and system familiarity during early trial implementation.

#### Chi-Square Analysis of Issue Incidence Across Centers

Chi-square testing across the three groups (Table 3) revealed no significant differences in CTPD, PDs, EDC/eCRF, and other issues (all *p* > 0.05). There was a statistically significant difference in the number of AE-related issues (χ^2^ = 10.682, *p* = 0.005).

Since the AE category differed among the three groups, post hoc pairwise comparisons were performed.

Chi-square analysis (Table 4) revealed a statistically significant difference in AE-related issues between center A and the other centers (χ^2^ = 8.320, *p* = 0.004). The incidence rate of AE-related issues per subject at center A was approximately 60.7% lower than that at the other centers (RR = 0.393; 95% CI: 0.276–0.559). No statistically significant differences were observed between center A and center B (*p* = 0.919) or between center B and the other centers (*p* = 0.073), although center B also had a lower incidence rate per subject compared with that at the other centers (RR = 0.416, 95% CI: 0.247–0.701).

These findings suggest that the implemented preventive measures had a measurable effect—most notably at center A—leading to more standardized AE identification, grading, and documentation.

Chi-square analysis revealed that compared with non-implementation, the implementation of preventive measures was associated with a statistically significant reduction in the incidence of AE-related issues at center A. Although the reduction observed at center B did not reach statistical significance in the direct comparison with center A, the average number of AE issues per subject at center B (2.33) remained substantially lower than that observed at the other centers (5.60).

Compared with the prior trial, the incidence of PDs at other centers decreased markedly in the subsequent trial, primarily because of enhanced subject management, as evidenced by the absence of cases with ≥10 PDs per subject.

### 3.6. Longitudinal Comparison of Center A Between the Two Trials

**Figure 3:**
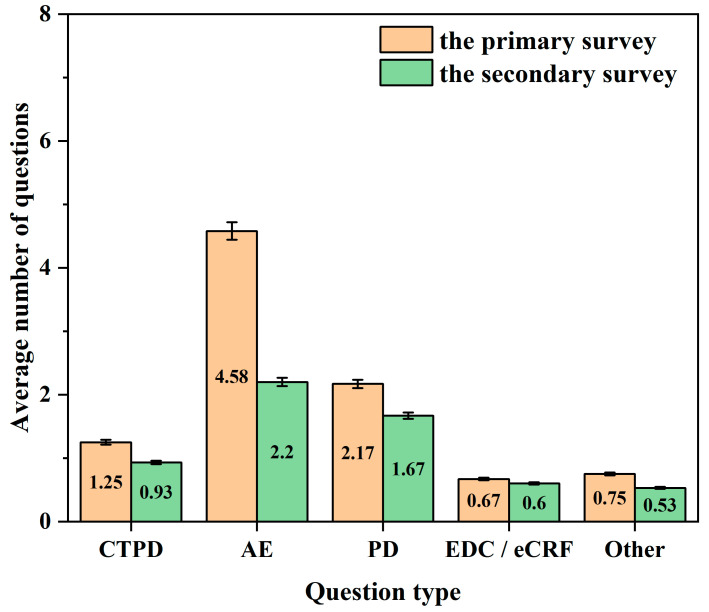
Comparative analysis of the average number of issues per subject by problem category at center A between the primary survey and the subsequent secondary survey. CTPD: Clinical trial process documentation; AE: Adverse event; PD: Protocol deviation; EDC/eCRF: Electronic data capture/electronic case report form.

A direct comparison of center A across the two surveys (Figure 3) demonstrated reductions in all five issue categories following the implementation of the preventive measures. Improvements were particularly pronounced in terms of AE documentation quality, PD occurrence, and CTPD completeness.

The results of this before–after comparison further support the effectiveness of preventive strategies and their adaptability for routine quality management in stroke-related clinical research.

### 3.7. Self-Controlled Concurrent Study at Center A

To minimize the influence of learning effects, we analysed and summarized the quality issues identified in another large-area cerebral infarction clinical trial conducted concurrently at Center A that did not implement preventive measures (Trial B). The number of issues across categories in Trial B was used as a control and compared with that in Trial A (the trial in which preventive measures were implemented) during the secondary survey.

Trial B was a phase II clinical trial; in total, 10 subjects were enrolled, and 90 issues were recorded. A comparison of the average number of issues per subject across categories between Trial A and Trial B is shown below.

**Figure 4:**
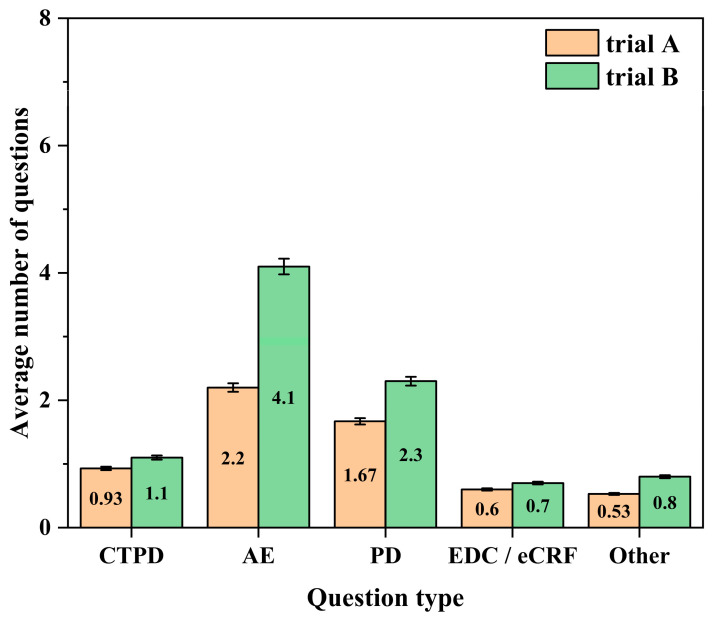
Comparative analysis of the average number of issues per subject by problem category between trial A and trial B at center A in the secondary survey. CTPD: Clinical trial process documentation; AE: Adverse event; PD: Protocol deviation; EDC/eCRF: Electronic data capture/electronic case report form.

A comparison of the self-controlled data at Center A (Figure 4) revealed that compared with Trial B, Trial A, in which preventive measures were implemented, exhibited reductions across all five issue categories, with the most pronounced improvement observed for AE-related issues. However, the magnitude of improvement was smaller than that observed in the longitudinal comparison of Center A between the two surveys, suggesting the presence of certain learning effects at Center A. Nevertheless, the preventive measures still demonstrated a preventive effect across various issue categories, particularly AE-related issues.

### 3.8. Construction of an Intervention Flow Model for Various Quality Issues

By analysing the effects of preventive measures at center A and center B, a cluster quantitative analysis of quality defects throughout the full trial cycle was conducted. The data flow perfectly mapped the design logic of the intervention system, confirming that the proposed intervention strategy possessed a high degree of scientific rigor, precision, and systemic comprehensiveness.

In the implementation of clinical trials, the standardized management of adverse event (AE) issues and the control of protocol deviations (PDs) directly determine the protection of subject rights and the core scientific value of clinical data. The model data indicated that investigators did not adopt a uniform, evenly distributed approach to intervention management but instead concentrated the most intensive intervention resources on the nodes with the highest AE issue flow. Through tool-based interventions, such as standardizing clinical judgement criteria and developing the AE Quick Reference Guide, frontline investigators were provided with standardized tools, substantially reducing the risk of underreporting and misreporting caused by subjective judgement variability. This strategy of “targeting the core pain points” with concentrated efforts demonstrated a high level of clinical management insight.

**Figure 5:**
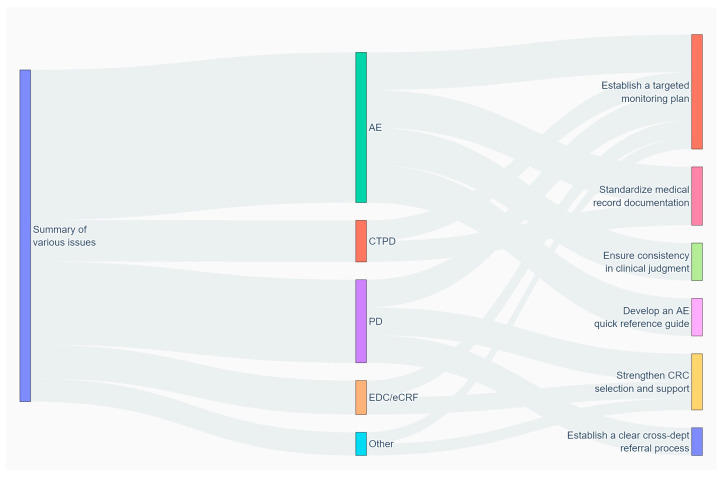
Intervention flow model for various quality issues. CTPD: Clinical trial process documentation; AE: Adverse event; PD: Protocol deviation; EDC/eCRF: Electronic data capture/electronic case report form.

The flow data (Figure 5) revealed that the establishment of a targeted monitoring plan served as the endpoint control for all five categories of quality issues, acting as an “absolute hub” within the intervention network. These findings hold significant academic and practical implications: they indicate that the intervention system not only relies on front-end standard development and tool support but also incorporates a robust dynamic correction closed loop at the back end. Whether issues arose from coordination challenges during cross-departmental referrals or data entry errors at the CRC level, they ultimately fell into the verification “filter” of targeted monitoring. This comprehensive, multidimensional systemic oversight provided a clinical trial with strong risk resistance and self-correction capabilities.

The design of the detailed pathways in this intervention model closely aligns with the core principles of a modern clinical quality management system (QMS). For instance, in addressing operational errors related to the EDC/eCRF and other issues, the intervention flow was precisely directed towards strengthening CRC screening and support. This reflects the research team’s keen recognition that the root cause of such issues was “insufficient human performance capacity at the execution level,” thereby addressing the problem by optimizing CRC resources. In addressing protocol deviations, the approach not only strengthened personnel support but also introduced mechanism optimization through the establishment of a clear cross-departmental referral process. This comprehensive collaborative ecosystem, encompassing personnel empowerment (CRC support), mechanism optimization (referral process), and tool support (quick reference guides)—a tripartite synergy of “people, process, and tools”—represents a key breakthrough for achieving homogeneous, high-quality execution in clinical trials.

## 4. Discussion

This study evaluated quality issues encountered in clinical trials for LHI and examined the impact of implementing targeted preventive measures. The findings demonstrate that structured, center-level quality interventions can meaningfully improve how trials are conducted, particularly in settings where disease severity and operational complexity pose substantial risks to protocol compliance.

Across centers, the overall incidence of quality issues in the subsequent secondary survey was notably lower than that in the primary survey, indicating improved operational control. Importantly, compared with the other centers, the two centers where preventive measures were implemented—center A and center B—exhibited consistently fewer issues per subject. This pattern was observed across multiple categories, suggesting that the interventions addressed both investigator-related and system-level contributors to quality risk.

The chi-square analysis provided further evidence of effectiveness. At center A, where preventive measures were implemented and the primary survey was conducted, there was a statistically significant reduction in AE-related issues compared with those at other centers. Given that AE documentation and assessment are among the most error-prone components of neurological clinical trials—owing to the dynamic progression of symptoms and high patient acuity—this targeted improvement is particularly meaningful. At center B, there was also a lower AE issue rate, although not statistically significant, likely because of its smaller sample size.

The results of a longitudinal comparison of center A across the two trials reinforced the effectiveness of the interventions. After the implementation of preventive measures, the number of issues per subject decreased from 9.42 to 5.93, representing a 37.0% reduction. Furthermore, a self-controlled study revealed that even in the presence of learning effects, preventive measures still demonstrated a preventive effect on the occurrence of various quality issues, particularly the incidence of AE-related issues. Improvements were observed across all five categories of metrics, indicating that the preventive measures produced broad, rather than category-specific, benefits. These results were similar to those of another study in which the implementation of structured quality improvement tools resulted in a 57.8% reduction in overall issue incidence [33]. Another study on the application of the PDCA cycle in the quality management of drug clinical trials revealed that after the implementation of PDCA cycle theory in 2019, the overall incidence of issues such as nonstandard case reports from documentation, protocol deviations, adverse event reporting problems, investigational product management issues, and informed consent problems was significantly lower (1.84%) than the 10.17% observed in 2018 (*p* < 0.05) [34]. Although the methodology differs and the magnitude of improvement in this study is relatively modest, it should be noted that the quality control in the large hemispheric infarction clinical trials addressed here inherently involves greater challenges. A multicenter standardization study on preclinical stroke research revealed that the complexity of stroke models, the urgency of the time window, and process differences in multicenter collaboration are the main sources of quality variability, which is highly consistent with the challenges faced in the present study [35]. Another review on selective enrolment issues in acute stroke trials also emphasized that stroke trials are substantially more difficult to manage in terms of quality control than conventional drug trials because of the urgency of the condition and the short decision-making window [36]. Nevertheless, a marked downward trend was observed specifically for AE-related issues, which represent one of the major problem categories. This suggests that proactive, process-oriented interventions—whether framed within Six Sigma or clinical-operations-specific frameworks—can substantially enhance quality and reduce variability in trial execution.

However, this study did not employ preventive measures at random levels in any of the involved centers. Therefore, the proposed preventive methods require further validation in larger-scale studies.

## 5. Conclusions

In this study, we proposed several preventive methods for improved quality control in large-area cerebral infarction clinical trials that can also benefit the management of stroke-related clinical trials. In detail, (1) centers with high patient acuity require tailored operational frameworks to support complete and timely assessments; (2) preventive measures such as standardized AE guides, structured documentation templates, and early, intensified monitoring appear particularly effective for minimizing documentation errors and protocol deviations; and (3) improvements are most pronounced when measures are implemented at the outset of a trial, underscoring the importance of early, prospective planning rather than reactive remediation. The effectiveness of these preventive methods requires further validation in other relatively large-scale clinical studies.

## Figures and Tables

**Table 1:** Categorization of quality issues.

Issue Category	Number of Issues at Center A	Number of Issues at the Other Centers
Clinical trial process documentation (CTPD)	15	35
Clinical data source verification	1	3
Subject screening and eligibility	4	5
Serious adverse event (SAE)/suspected unexpected serious adverse reaction (SUSAR)/adverse event of special interest (AESI) recording and reporting	2	9
AE	55	172
Protocol deviation (PD)	26	209
Electronic data capture/electronic case report form (EDC/eCRF)	8	18
Management and documentation of biological	1	4
samples		
Monitoring	1	3
Total	113	458

CTPD: clinical trial process documentation; AE: adverse event; PD: protocol deviation; EDC/eCRF: electronic data capture/electronic case report form; SAE: serious adverse event; SUSAR: suspected unexpected serious adverse reaction; AESI: adverse event of special interest.

**Table 2:** Analysis of issue frequency in the primary survey.

Group	Total Subjects Enrolled	CTPD	AE	PD	EDC/eCRF	Other
Center A	12	15	55	26	8	8
Other centers	28	35	172	209	18	24
χ^2^		0	0.603	10.292	0.004	0.222
*p*		1	0.438	0.001	0.947	0.638

CTPD: clinical trial process documentation; AE: adverse event; PD: protocol deviation; EDC/eCRF: electronic data capture/electronic case report form.

**Table 3:** Test of issue frequency in the subsequent secondary survey.

Group	Total Subjects Enrolled	CTPD	AE	PD	EDC/eCRF	Other
Center A	15	14	33	25	9	8
Center B	6	5	14	10	6	4
Other centers	84	93	470	249	57	63
χ^2^		0.357	10.682	3.665	0.536	0.546
*p*		0.836	0.005	0.160	0.765	0.761

CTPD: clinical trial process documentation; AE: adverse event; PD: protocol deviation; EDC/eCRF: electronic data capture/electronic case report form.

**Table 4:** Chi-square test results for AE issue frequency between groups.

Comparison Group	Group	Issues Recorded	Total Subjects Enrolled	χ^2^	*p*	RR	95% CI
Center A vs. Center B	Center A	33	15	0.1	0.919	0.944	(0.506–1.762)
	Center B	14	6				
Center A vs. the other Centers	Center A	33	15	8.320	0.004	0.393	(0.276–0.559)
	Other centers	470	84				
Center B vs. the other Centers	Center B	14	6	3.214	0.073	0.416	(0.247–0.701)
	Other centers	470	84				

## Data Availability

The raw data supporting the conclusions of this article will be made available by the authors upon request.

## Abbreviations

CTPD	clinical trial process documentation
AE	adverse event
PD	protocol deviation
EDC/eCRF	electronic data capture/electronic case report form
LHI	large hemispheric infarction
SAE	serious adverse event
SUSAR	suspected unexpected serious adverse reaction
AESI	adverse event of special interest
CRC	clinical research coordinator
